# Immune Checkpoint Inhibitors-Related Thyroid Dysfunction: Epidemiology, Clinical Presentation, Possible Pathogenesis, and Management

**DOI:** 10.3389/fendo.2021.649863

**Published:** 2021-06-10

**Authors:** Ling Zhan, Hong-fang Feng, Han-qing Liu, Lian-tao Guo, Chuang Chen, Xiao-li Yao, Sheng-rong Sun

**Affiliations:** ^1^ Department of Breast and Thyroid Surgery, Renmin Hospital of Wuhan University, Wuhan, China; ^2^ Department of Breast Surgery, Thyroid Surgery, Huangshi Central Hospital, Affiliated Hospital of Hubei Polytechnic University, Edong Healthcare Group, Huangshi, China

**Keywords:** immune checkpoint inhibitors, immune-related adverse events, thyroid dysfunction, epidemiology, clinical manifestations, pathogenesis, management

## Abstract

Immune checkpoint inhibitors (ICIs) are a group of drugs employed in the treatment of various types of malignant tumors and improve the therapeutic effect. ICIs blocks negative co-stimulatory molecules, such as programmed cell death gene-1 (PD-1) and its ligand (PD-L1) and cytotoxic T-lymphocyte-associated antigen-4 (CTLA-4), reactivating the recognition and killing effect of the immune system on tumors. However, the reactivation of the immune system can also lead to the death of normal organs, tissues, and cells, eventually leading to immune-related adverse events (IRAEs). IRAEs involve various organs and tissues and also cause thyroid dysfunction. This article reviews the epidemiology, clinical manifestations, possible pathogenesis, and management of ICIs-related thyroid dysfunction.

## Introduction

The immune system plays an important role in the occurrence, development, and prognosis of most tumors and forms a specific tumor immune microenvironment. The immune system can recognize, kill, and resist tumor cells. However, tumor cells can escape the killing or clearance effect of the immune system through various escape mechanisms. For example, the immune checkpoint pathway could be activated to inhibit the anti-tumor immune response (1). Some cell surface receptors play a significant role in the process. Programmed cell death gene-1 (PD-1) and its ligand (PD-L1), known as negative co-stimulatory molecules, are the second signal of T cell activation in cellular immune response (2). Cytotoxic T-lymphocyte-associated antigen-4 (CTLA-4) is another inhibitory receptor of active T cells by high-affinity binding to natural B7 family ligands, which plays a similar role (3). They work together with the first signal to inhibit T cells and regulate the immune response. The original function of the immune checkpoint is to maintain immune homeostasis and prevent autoimmunity (**Box 1**) (2, 3). However, those pathways are activated to escape the cytotoxic T-lymphocyte cell (CTL)-mediated immune killing effect in most malignant tumor cells (4, 5).

Box 1 **PD-1/PD-L1 and CTLA-4 play a role as the immune checkpoint.**

The receptor on the surface of the T cell (TCR) binds to an antigen, acting as the first signal to activate T cells (1). The second signal of T cell activation in cellular immunity is composed of costimulatory molecules on the surface of T cells, antigen-presenting cells (APCs), and target cells. There are numerous costimulatory molecules on the T cell surface, including positive and negative costimulatory molecules such as CD28, PD-1, and CTLA-4 (2, 3, 19, 21). PD-L1 is found on tumor cells and APCs, such as B cells, dendritic cells (DCs), and macrophages (2, 21). PD-1 binds to PD-L1, working together with the first signal to inhibit T cells and regulate the immune response (2). CTLA-4, which is similar to its homologous stimulatory receptor CD28, combines with natural B7 family ligands, CD80 and CD86, and exerts an immunomodulatory role (3). To conclude, the PD-1/PD-L1 pathway and CTLA-4 play a vital role as immune checkpoints, which interact with positive costimulatory molecules, so that immune response can start effectively, play a role properly, and terminate in time (**Figure 2**).

Some immunotherapeutic agents can block those intercellular signal transductions, and thus eliminate the inhibitory effect of T cells, which restore the anti-tumor response (6). In recent years, immune checkpoint inhibitors (ICIs) are reported to be novel agents for the treatment of malignant tumors, which show promising therapeutic effects and potential (7–9). Although ICIs are often described as well tolerated, sometimes, they still produce inevitable immune-related adverse events (IRAEs). ICIs activate the immune system, affect normal organ tissues, and lead to cell death in addition to targeting tumor cells, eventually leading to IRAEs (6). IRAEs involve various organs and systems of the whole body and also cause thyroid dysfunction, which needs clinical attention (10–14).

**Figure 1:**
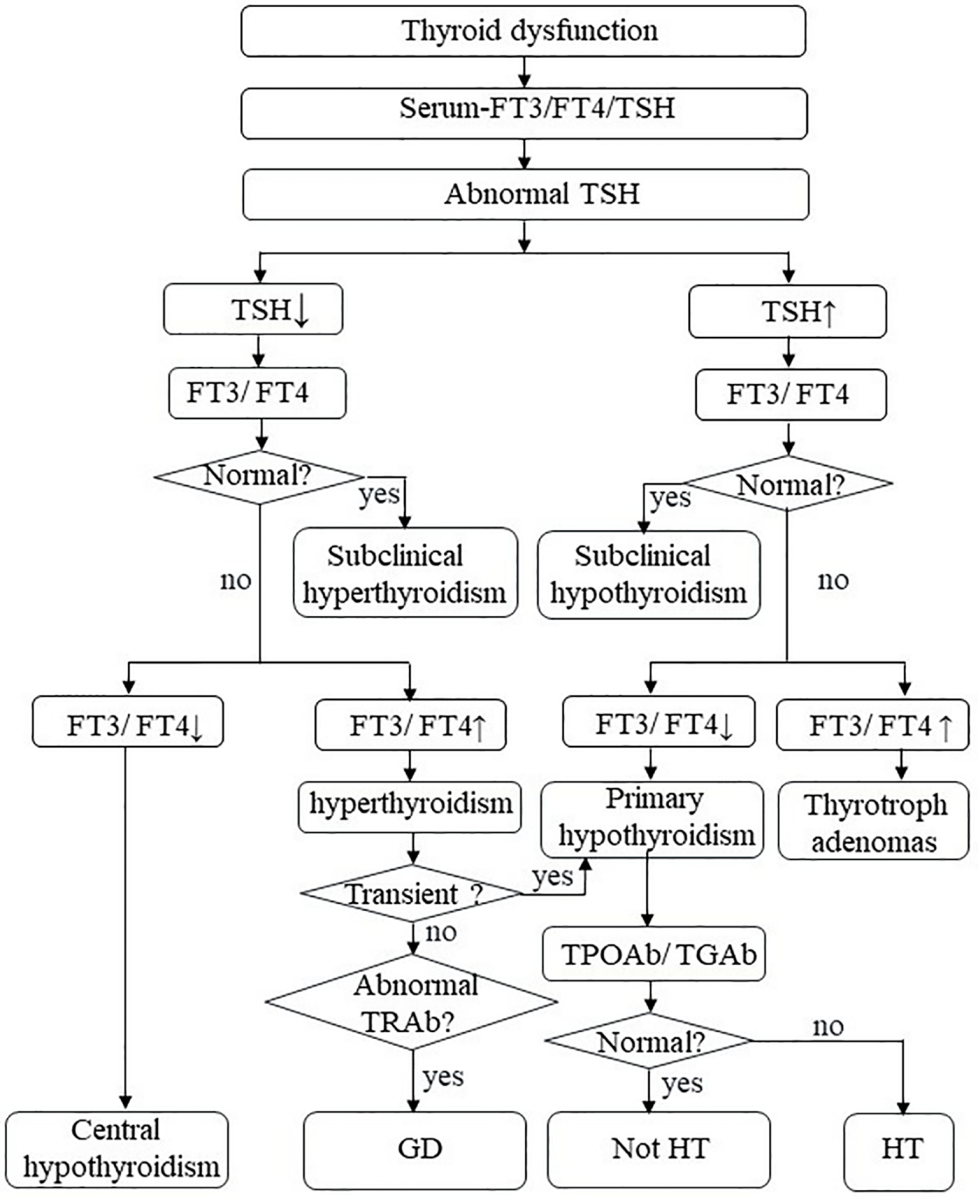
Thyroid dysfunction. FT3, free triiodothyronine; FT4, free thyroxine; TSH, thyroid-stimulating hormone; TPO-Ab, thyroperoxidase antibodies; TG-Ab, thyroglobulin antibody; TRAb, TSH receptor antibodies; HT, Hashimoto’s thyroiditis; GD, Graves’ disease.

Thyroid dysfunction is a common pathological state of thyroid hormone disorder, most commonly hypothyroidism (15). It needs active surveillance and treatment; otherwise, severe thyroid dysfunction may seriously affect health in some cases (16, 17). The specific mechanism of hypothyroidism is still unclear and warrants further laboratory and clinical exploration. Currently, the diagnosis of thyroid dysfunction depends primarily on the identification of biochemical indicators due to a lack of special symptoms (**Figure 1**) (18). Although thyroid dysfunction is mild among all IRAEs, they have considerable morbidity (19, 20). Better characterization of thyroid IRAEs and their underlying mechanisms could improve clinical identification, management, and care of these patients and assist in choosing a more effective treatment.

## ICIs and IRAEs

The common ICIs, approved by the US Food and Drug Administration (FDA), include Ipilimumab for anti-CTLA-4 therapy (5), Nivolumab, Pembrolizumab and Cempilimab for anti-PD-1 therapy (22–25), Durvalumab, Atezolizumab and Avelumab for anti-PD-L1 therapy (**Table 1**) (10, 26–28). Currently, ICIs have been widely used in cutaneous squamous cell carcinoma (cSCC), triple-negative breast cancer (TNBC), urothelial carcinoma (UTUC), squamous cell carcinoma of the anal canal (SCCA), malignant melanoma, renal cell carcinoma (RCC), non-small cell lung cancer (NSCLC), small cell lung cancer (SCLC), lung adenocarcinoma (LUAD) etc. (25, 27–41).

**Table 1:** Comparison of ICIs-related thyroid dysfunction

Author & year	Study type	Target tumor	ICIs	Thyroid dysfunction	Incidence	
					Any Grade	Grade 3-5
				(%)	(%)	
Migden et al., 2020 (24)	RCT	cSCC	Cempilimab	Hypothyroidism	10.0	0
Migden et al., 2018 (25)	RCT	cSCC	Cempilimab	Hypothyroidism	8.0	0
Loibl et al., 2019 (26)	RCT	TNBC	Durvalumab	Hypothyroidism	7.6	0
				Hyperthyroidism	9.8	0
Mittendorf et al., 2020 (27)	RCT	TNBC	Atezolizumab	Hypothyroidism	6.7	0
				Hyperthyroidism	3.0	0
Powles et al., 2020 (28)	RCT	UTUC	Avelumab	Hypothyroidism	11.6	0.3
Morris et al., 2017 (29)	RCT	SCCA	Nivolumab	Hypothyroidism	6.0	3.0
Wolchok et al., 2017 (30)	RCT	melanoma	Ipilimumab	Hypothyroidism	5.0	0
				Hyperthyroidism	1.0	0
			Nivolumab	Hypothyroidism	11.0	0
				Hyperthyroidism	4.0	0
			Nivolumab + Ipilimumab	Hypothyroidism	17.0	<1.0
				Hyperthyroidism	11.0	1.0
Ascierto et al., 2020 (31)	RCT	melanoma	Nivolumab	Hypothyroidism	<1.0	0
			Ipilimumab		<1.0	0
Eggermont et al., 2018 (32)	RCT	melanoma	Pembrolizumab	Hypothyroidism	14.3	0
				Hyperthyroidism	10.2	0.2
				Transient thyroiditis	3.1	0
Motzer et al., 2018 (33)	RCT	RCC	Nivolumab + Ipilimumab	Hypothyroidism	16.0	<1.0
Koshkin et al., 2018 (34)	RCT	RCC	Nivolumab	Hypothyroidism	7.0	0
McDermott, et al., 2021 (35)	RCT	RCC	Pembrolizumab	Hyperthyroidism	5.5	0
Osorio et al., 2017 (9)	RCT	NSCLC	Pembrolizumab	Hypothyroidism	8.0	NA
				Transient thyroiditis	13.0	NA
Hellmann et al., 2019 (36)	RCT	NSCLC	Nivolumab + Ipilimumab	Hypothyroidism	12.0	<1.0
Hellmann et al., 2018 (37)	RCT	Lung Cancer	Nivolumab + Ipilimumab	Hypothyroidism	11.6	0.3
			Nivolumab		6.4	0.3
Patel et al., 2020 (38)	RCT	Neuroendocrine Neoplasms	Ipilimumab + Nivolumab	Hypothyroidism	31.3	0

ICIs, immune checkpoint inhibitors; RCT, a randomized controlled trial; cSCC, cutaneous squamous cell carcinoma; TNBC, triple-negative breast cancer; UTUC, urothelial carcinoma; SCCA, squamous cell carcinoma of the anal canal; RCC, renal cell carcinoma; NSCLC, non-small cell lung cancer; NA, not available.

ICIs are often accompanied by IRAEs, including hypophysitis, thyroid dysfunction, and autoimmune diabetes, which can occur alone or concurrently (10–12, 23, 41). So far, numerous articles have reviewed the incidence rate of IRAEs, among which thyroid IRAEs was found to be the most common (19, 20). However, it is less likely to accurately predict the system or organ to be affected by IRAEs. Therefore, more prospective studies are needed to explore the predictive biomarkers of IRAEs.

## Specific Effects of IRAEs on the Thyroid

### Epidemiology

Some scholars have reported that most patients on ICIs for malignancies are at risk of developing thyroid dysfunction. Thyroid IRAEs present mainly as hypothyroidism, hyperthyroidism, and transient thyroiditis (10, 13, 14, 22). Transient thyroiditis was diagnosed as noticeable hyperthyroidism or subclinical hyperthyroidism at the time of diagnosis and subsequently progressing to hypothyroidism (22). The latest review and meta-analyze have reported high thyroid IRAEs frequencies, especially relatively high risk for hypothyroidism (42). Notably, ICIs-related thyroid dysfunction incidences lie on the type of malignant tumor and ICIs employed (**Table 1**). Stelmachowska-Banas et al. (42) summarized that combination therapy has been associated with the highest estimated incidence of high thyroid dysfunction frequencies, ranging from 8.0 to 16.4%, remarkably higher than monotherapy with anti-PD-1 drugs (2.8-8.5%) or anti-PD-L1 drugs (0.6-6.0%) or anti-CTLA-4 (0.2-5.2%). The combination of multiple immunotherapies can increase the risk of thyroid dysfunction (23, 43–46). What’s more, the incidence of hypothyroidism, hyperthyroidism, and thyroiditis was statistically significant between different drugs (47, 48). Previous researchers found that the probability of thyroid dysfunction in the anti-PD-1 treatment group was higher than that in the anti-PD-L1 and anti-CTLA-4 treatment group (45, 49). Furthermore, although both Nivolumab and Pembrolizumab are anti-PD-1 drugs, patients using the former are more likely to develop hypothyroidism, whereas those using the latter are more likely to develop hyperthyroidism (**Table 1**) (49). Notably, the type of ICIs-related thyroid dysfunction was not completely identical among different tumors. We can be implied from several prospective studies that malignant melanoma and TNBC patients have a certain risk of hyperthyroidism incidence (26, 27, 30, 32), while malignant melanoma and NSCLC patients also have a risk of transient thyroiditis (9, 32). To concluded, patients on combination therapy were significantly more prone to develop thyroid dysfunction than those receiving monotherapy. And patients treated with anti-CTLA-4 drugs had a significantly lower risk for thyroid dysfunction compared to those with anti-PD-1 and anti-PD-L1.

### Clinical Manifestations

The biochemical behavior of thyroid dysfunction is different between tumor types as well as immunosuppressive therapy (**Table 1**). Ohara et al. (22) reported that a 69-years-old patient with LUAD developed painless thyroiditis during a 3-month nivolumab treatment. The patient had a mild and soft goiter but had no symptoms of thyrotoxicosis or exophthalmos. She did not present any fever or pain. Serum-free thyroxine (FT4) was elevated and thyroid-stimulating hormone (TSH) was decreased; shortly after, primary hypothyroidism began to appear. Another patient had the same thyroid disorder after 6 months of treatment with nivolumab for melanoma (50). An 85-year-old male suffered hypothyroidism coexisting with various autoimmune diseases after the administration of pembrolizumab for advanced melanoma (23). In another case, after 4 months of Durvalumab immunotherapy, the level of FT4 decreased and that of TSH increased in a 49 years old female patient with LUAD (10). However, thyroperoxidase antibodies (TPO-Ab) and thyroglobulin antibody (TG-Ab) were negative (22, 50). In summary, most of the patients were diagnosed with transient thyroiditis or hypothyroidism during immune checkpoint blockade, which can be verified in many retrospective studies (51, 52). Even though many studies have viewed the highest incidence rate of hypothyroidism, thyroiditis is not uncommon (51, 53, 54). According to the cause of hypothyroidism, among which ICIs-related hypothyroidism is categorized as the primary hypothyroidism. Primary hypothyroidism is defined as TSH level higher than the reference range and FT4 level lower than the reference range (15). In turn, central hypothyroidism is defined as low or low-to-normal TSH level and a disproportionately low FT4 level, owing to dysfunction of the hypothalamus or the pituitary gland, or both (55). Generally, most patients present with primary hypothyroidism, and only a few cases with hypothalamic or pituitary dysfunction have secondary central hypothyroidism (**Figures 1** and **3**) (18, 56). Patients may be asymptomatic or only show non-specific symptoms, such as fatigue, dizziness, weight changes, and emotional or behavioral changes (18). Thyroid disorders are often neglected because their presentation is often inconspicuous and only a few patients have thyroid storms (16). It can also be easily deduced from the aforementioned literature that the median time from the beginning of drug commencement to the development of thyroid dysfunction varies in different immunotherapies. Thyroid dysfunction has been reported to mostly occurs in 5-36 weeks after immunotherapy (13, 48, 57). In a retrospective study, the median occurrence time and the duration time of thyroiditis was 5.3 weeks (range 0.6-19.6 weeks) and 6 weeks (range 2.6-39.7 weeks), and the median occurrence time of hypothyroidism was 10.4 weeks (range 3.4-48.7 weeks) (54). Although a few patients develop permanent hypothyroidism, most of them can be relieved after suspending immunotherapy or undergoing thyroid hormone replacement therapy (22, 23). Finally, the recovery time of thyroid dysfunction among patients with combination therapy was significantly longer than that of patients with monotherapy (13, 14).

To conclude, patients can present with hypothyroidism or transient thyroiditis during the commencement of ICIs (**Table 1**). However, these patients are mostly detected during routine hormone monitoring because of a lack of clinical symptoms. The dynamic changes of free triiodothyronine (FT3), FT4, and TSH can be detected but there are few reports of positive TPO-Ab and TG-Ab. Additionally, based on the Common Terminology Criteria for Adverse Events (CTCAE) Version 5.0, recommended by the National Cancer Institute (58), thyroid IRAEs are mostly graded from level 1 to 3 (**Tables 1** and **2**) (9, 42, 45, 59).

**Table 2:** Thyroid IRAEs grade in the CTCAE Version 5.0.

Thyroid IRAEs					
Term	Grade 1	Grade 2	Grade 3	Grade 4	Grade 5
**Hypothyroidism**	Asymptomatic; clinical or diagnostic observations only;intervention not indicated	Symptomatic; thyroid replacement indicated; limiting instrumental ADL	Severe symptoms; limiting self-care ADL; hospitalization indicated	Life-threatening consequences; urgent intervention indicated	Death
**Hyperthyroidism**	Asymptomatic; clinical or diagnostic observations only; intervention not indicated	Symptomatic; thyroid suppression therapy indicated; limiting instrumental ADL	Severe symptoms; limiting self-care ADL; hospitalization indicated	Life-threatening consequences; urgent intervention indicated	Death
**Thyroiditis**	Asymptomatic; clinical or diagnostic observations only; intervention not indicated	Symptomatic; thyroid suppression therapy indicated; limiting instrumental ADL	Severe symptoms; limiting self-care ADL; hospitalization indicated	Life-threatening consequences; urgent intervention indicated	Death

Hypothyroidism: a disorder characterized by a decrease in the production of thyroid hormone by the thyroid gland.

Hyperthyroidism: a disorder characterized by excessive levels of thyroid hormone in the body. Common causes include an overactive thyroid gland or thyroid hormone overdose.

Thyroiditis: a disorder characterized by transiently obvious hyperthyroidism or subclinical hyperthyroidism and subsequently hypothyroidism.

IRAEs, immune-related adverse events.

## Mechanism of ICIs-Related Thyroid Dysfunction

The underlying mechanism for ICIs-related thyroid dysfunction remains unknown. In terms of clinical presentation, hypothyroidism or hypothyroidism after transient thyrotoxicosis is the most common and consistent characteristic of patients with ICIs. Thyroid IRAEs seem to overlap with that of autoimmune thyroid diseases (AITDs), such as Graves’ disease (GD) (60), Hashimoto’s thyroiditis (HT) (61). The thyroid gland is known to be more susceptible to autoimmune attacks than any other organ (62). Hypothyroidism often occurs after hemithyroidectomy, radioiodine therapy, and neck radiotherapy (15, 63). Whether thyroid IRAEs have the same mechanism as AITDs, warrants further elucidation.

### Link Between the Immune System and HT and GD

**Figure 2:**
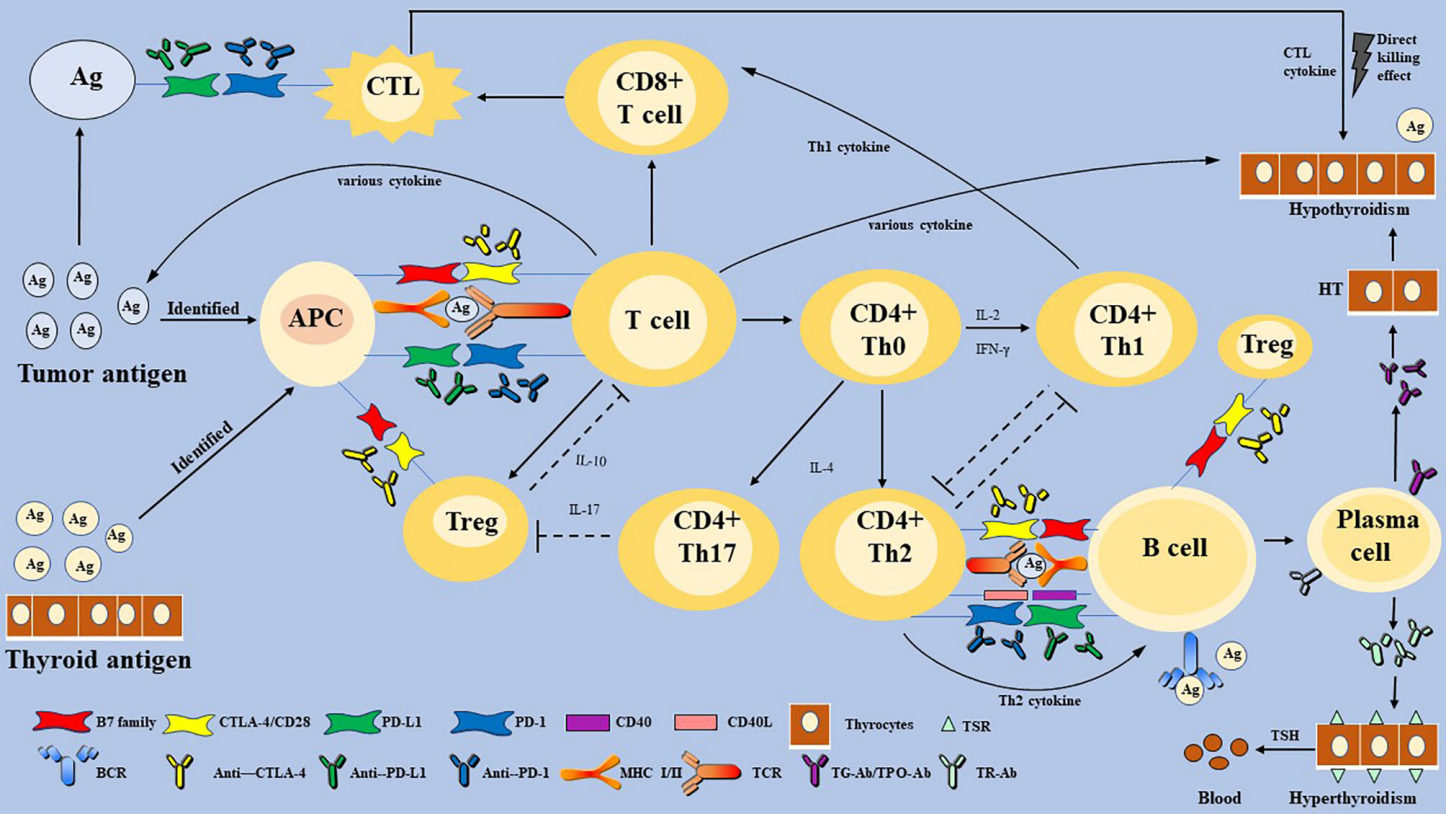
The proposed mechanism of immune checkpoint inhibitors-related thyroid dysfunction. Thyroid IRAEs may involve T and B-lymphocytes, multiply cytokines, and diverse factors. Immune checkpoints are activated to escape the immune killing and clearance effect in most malignant tumor cells. Some immunotherapeutic agents can eliminate the inhibitory effect of T cells, which restore the anti-tumor response. However, activation of the immune system can also affect normal organ tissues, and lead to cell death, eventually leading to organ IRAEs. Thyroid IRAEs present mainly as hypothyroidism, hyperthyroidism, and transient thyroiditis, seem to overlap with AITDs. HT and GD are AITDs that cause hypothyroidism and hyperthyroidism, respectively. HT is caused by impaired immune tolerance of autoantigens, the destruction of thyroid cells. The pathogenesis of HT is considered to be a complex autoimmune process involving various activate and infiltrate T lymphocytes, B lymphocytes, and various cytokines. Then a cellular immune response and humoral immune response are induced, leading to direct thyroid injury and further thyroid antigen exposure. The main pathogenesis of GD can be understood as the combination of TSH receptor and TR-Ab secreted and released by Th2 cell-dependent B cells. Immune checkpoints are proposed to play a role in inhibiting the autoimmune process by inhibiting various immune cells. Whether thyroid IRAEs have the same mechanism as AITDs, warrants further elucidation. PD-1, programmed cell death gene-1; PD-L1, programmed cell death gene-1 ligand; CTLA-4, cytotoxic T-lymphocyte-associated antigen-4; MHC, major histocompatibility complex; Th cells, helper T cells; CTL, cytotoxic T lymphocyte cell; Treg, T-regulatory cells; APCs, antigen-presenting cells; TSH, thyroid-stimulating hormone; TPO-Ab, thyroperoxidase antibodies; TG-Ab, thyroglobulin antibody; TRAb, TSH receptor antibodies; AITDs, autoimmune thyroid diseases; HT, Hashimoto’s thyroiditis; GD, Graves’ disease.

HT, widely seen as the common cause of hypothyroidism (15), is caused by impaired immune tolerance of autoantigens, the destruction of thyroid cells (64, 65). The pathogenesis of HT is considered to be a complex autoimmune process. Various T lymphocytes (**Box 2**) activate and infiltrate thyroid follicular cells, and then induce a cellular immune response leading to direct thyroid injury and further thyroid antigen exposure (65). However, B lymphocytes participate in humoral immune response and secrete specific TPO-Ab and TG-Ab against thyroid auto-antigen (61, 65). Besides, natural killer (NK) cells, macrophages, and various cytokines, such as Type 1 T helper (Th1) cytokines (interleukin-2 (IL-2) and interferon-gamma (IFN-γ)), T-regulatory cells (Treg) cytokine (IL-10), and Th17 cytokine (IL-17), participate in the autoimmune process (62). Meanwhile, CTL and Th1-mediated immune responses play a leading role in the development of autoimmune diseases (43). However, the body does not allow the immune system’s unrestricted self-attack on the thyroid gland. Treg and PD-1 pathways may be triggered and activated because of persistent autoimmunity. Treg has an inhibitory effect on autoimmunity, which is inhibited in HT until it is reactivated by uncontrolled autoimmunity (62). Álvarez-Sierra et al. (67) detected the expression of PD-1 in peripheral blood and thyroid gland among HT patients and found that the expression of CD4^+^ and CD8^+^ PD-1 positive T cells in the thyroid gland was increased. In the background of lymphocytic thyroiditis and hyperthyroidism, PD-L1 expression in benign follicular epithelial cells was also increased (67). Although the PD-1/PD-L1 pathway cannot stop the autoimmune reaction, it can inhibit the autoimmune response by inhibiting T cells. However, whether it achieves the effect of complete inhibition of disease or not remains unknown. It is reasonable to speculate that HT does not progress as rapidly as acute thyroiditis, which may be due to the negative regulatory effect of Treg and the PD-1/PD-L1 pathway (**Figure 2**).

Box 2 **T cells and human leukocyte antigen (HLA).**

T cells can be divided into naïve T cells, effector T cells, and memory T cells based on the activation stage. T cells can also be divided into CD4^+^ and CD8^+^ T cells. Further, T cells are divided into helper T (Th) cells, CTL, and Treg based on their functions. There exist a Th1/Th2 balance that transforms depending on the status of the immune response (64). T cells promote organ and tissue autoimmunity mainly through the following ways: activated T cells proliferate and differentiate, then transform into effector T cells, such as Th1 and CTL; Th2 cell-dependent B cells produce and secrete auto-antibodies; various inflammatory factors (21, 64). Furthermore, tissue damage in turn gives rise to further exposure of tissue self-antigen, which leads to more active T cells in the positive feedback loop (64). Then Treg, immune checkpoint, and other inhibitory pathways will be induced to eliminate self-immune (**Figure 2**) (21).

HLA gene complex, also called the major histocompatibility complex (MHC), is closely associated with immune response (66). With a complex structure, HLA gene regions are mainly divided into class I and class II, which are both directly involved in the activation and differentiation of T cells and regulation of adaptive immune response by binding to a specific antigen peptide (**Figure 2**) (21). HLA I is distributed on the surface of all nucleated cells, but HLA II only expresses surfaces specific cells, such as activated T cells and professional APCs. HLA II binds to antigen peptides and then to CD4 Th T cells receptor to accurately recognize Th T cells (21).

GD is an AITDs that causes hyperthyroidism. Its main pathogenesis can be understood as follows: the combination of TSH receptor on the thyroid cells surface and TSH receptor antibody (TR-Ab) secreted and released by Th2 cell-dependent B cells results in thyroid cell damage and a series of symptoms of hyperthyroidism caused by the release of thyroid hormone. Interestingly, hypothyroidism does not occur in patients with GD because Th2 is dominant (**Figure 2**) (60, 64).

### Link Between the Immune System and ICIs-Related Thyroid Dysfunction

#### Role of T Cell-Mediated Cellular Immune in Thyroid Dysfunction

Until now, the immune system activated by ICIs not only targets tumor cells but also leads to the death of normal organs, tissues, and cells, which is recognized as the most possible mechanism. Numerous studies have reported that increasing infiltrating CD4+ and CD8+ T cells represent a higher response rate and a better clinical outcome of ICIs because it also represents the activity of anti-tumor immunity (7–9, 68). Intriguingly, previous studies reported that increased circulating CD4^+^ and CD8^+^ T cells also presented a relatively higher incidence of IRAEs (69). However, a large amount of clinical and experimental data is needed to confirm the authenticity of this phenomenon.

ICIs may trigger T cell-mediated pathways that induce subsequent thyroid dysfunction (65, 70). Generally, anti-CTLA-4 treatment is more likely to trigger IRAEs than anti-PD-1 or PD-L1 treatment, because anti-CTLA-4 is more likely to lead to extensive T cell activation but blocking PD-1 or PD-L1 is likely to trigger pre-existing CD8^+^ T cell activation (6). However, in terms of thyroid IRAEs, the probability of anti-PD-1 or anti-PD-L1 agents was higher than that of anti-CTLA-4 agents. Additionally, blocking PD-1 is more likely to lead to the activation of pre-existing CD8^+^ T cells than PD-L1 and CTLA-4 inhibition (1), which also well explains why thyroid dysfunction in the anti-PD-1 treatment group was higher than that of the anti-PD-L1 and anti-CTLA-4 treatment group (45, 49). Besides, several patients receiving immunosuppressive therapy showed symptoms of hyperthyroidism before hypothyroidism (9). It is suspected that GD occurs first, and then thyroid cell antigen is exposed, which leads to autoimmunity and hypothyroidism because Th1 cells are dominant. Treg plays a role in the inhibitory effect through cell-cell contact and secreting a regulatory cytokine IL-10. Some studies have shown that a higher baseline IL-10 level can improve anti-PD-1 therapy response, indicating that PD-1 is involved in regulating the proliferation and differentiation of Treg (71). ICIs may cause the loss of Treg energy, inducing self-immune on the thyroid (72, 73). Taken together, T cell-mediated cellular immune is the main cause of thyroid IRAEs (**Figure 2**).

#### Role of Humoral Autoimmune Response in Thyroid Dysfunction

It is unclear whether PD-1 blockade induces B cell-mediated humoral autoimmune response. Whether patients with ICI-induced hypothyroidism have positive auto-antibodies is an unknown problem (74). Several case reports found that there were negative auto-antibodies among patients with the treatment of ICIs, although thyroid dysfunction occurred (22, 50). A few data indicate that blocking PD-1 induces T cell-dependent B cells to produce and secrete auto-antibodies and the presence of thyroid auto-antibodies and an early increase in serum thyroglobulin (Tg) levels may result in an increased risk of thyroid IRAEs (9, 71, 75, 76). However, it remains to be determined whether there are any specific risks in subgroups with previous subclinical autoimmune thyroid disease (77). There was also another notion that thyroid auto-antibodies result from humoral immune response to release thyroid antigens in the process of destructive thyroiditis. Of greatest interest, a single-center, retrospective cohort study conducted by Delivanis et al. (65) showed that a minority of the patients had positive TPO-Ab among patients with thyroid IRAEs. However, Delivanis and colleagues (65) did not suspect that the mechanism of thyroid destruction is related to thyroid auto-antibodies. Therefore, whether auto-antibodies are the reason for thyroid dysfunction or the result of destructive thyroiditis when applying ICIs remains controversial, which may be the focus of future research (**Figure 2**). Moreover, Delivanis et al. (65) reported that NK cells or monocyte-mediated pathways may be involved in thyroid IRAEs due to the elevated HLA class (**Box 2**) surface expression in CD56^+^CD16^+^ NK cells and CD14^+^CD16^+^ monocytes, which needs data to ascertain its credibility.

#### Role of Individual Genetic Susceptibility in Thyroid Dysfunction

It is worth mentioning that autoimmune diabetes and AITDs, are associated with genetic susceptibility associated with overexpression of HLA-DR (human leukocyte antigen-DR isotype) (66, 78–80). More interestingly, hypothyroidism is more prevalent in patients with autoimmune diseases, such as type 1 diabetes and autoimmune gastric atrophy, and sometimes occurs as part of various autoimmune endocrine diseases (80). This phenomenon also exists in people who employ ICIs to treat malignant tumors (23). The mechanisms by which hypothyroidism may be linked to systemic autoimmune diseases have not yet been fully understood (64). ICIs may change the expression of HLA-DR, increasing the abnormal activation of T cells and thyroid autoimmunity susceptibility (**Figure 2**). Delivanis et al. (65) found that macrophage activation by up-regulating HLA-DR may be a potential mechanism of pembrolizumab-induced thyroiditis. Krieg et al. (77) have reported that the frequency of CD14^+^CD16^−^ HLA-DR^hi^ monocytes are a strong indicator for progression-free survival (PFS) and overall survival (OS) of anti-PD-1 immunotherapy.

#### Role of Various Cytokines in Thyroid Dysfunction

Besides T and B lymphocytes, various cytokines play an essential role in the development of thyroid disorders (**Figure 2**) (62). Firstly, a higher level of IL-2 can not only induce the binding between HLA-II with thyroid cell autoantigen, but also promote the killing effect of CD8^+^ CTL on the thyroid (71, 81). Krieg et al. (77) found that the number of CD4^+^ Th1 that express IFN-γ and IL-2 increased after anti-PD-1 treatment, indicating that PD-1 and PD-L1 are involved in the inhibition of T cell proliferation and the production of pro-inflammatory Th1 cytokines, including IFN-γ and IL-2. Kurimoto et al. (71) measured the changes of various cytokines before and after the treatment of ICIs and found that an increase of IL-2 and a decrease of granulocyte colony-stimulating factor (G-CSF) were seemly correlated with thyroid IRAEs. Th2 cytokine has a strong positive correlation with G-CSF, whose decrease may be related to the decrease of Th2 cytokine activity, which also indicates the increase of Th1 dominance in thyroid IRAEs (71). In summary, it is a plausible suspicion that Th1 cytokines (IFN-γ and IL-2) are involved in thyroid autoimmunity through blocking PD-1 and PD-L1. The decrease of IL-10 may be related to the increase of TPO-Ab, suggesting the loss of Treg energy and the development of thyroid IRAEs. Additionally, the toxicity mediated by IL-17 has been shown to contribute to anti-CLTA-4 induced enterocolitis, which also suggested the loss of Treg energy.

#### Role of Aging and Gender in ICIs Thyroid Dysfunction

Aging itself is conducive to an increased incidence of autoimmune diseases and malignant tumors because of immune function disorder and remodeling of the immune microenvironment (82, 83). The expression of PD-L1 is a critical mechanism by which aging tissues prevent their reactive T cells from infinitely participating in autoimmunity (2, 23). Some scholars believe that the immune system activated by ICIs is more likely to lead to thyroid self-immunity among the elderly (25). However, in an ICIs safety study among elderly patients with NSCLC, aging was not a high-risk factor for IRAEs (84). Sex hormones are also involved in the regulation of the immune system (85) but the relationship between gender and IRAEs remains unknown because of numerous contrary conclusions (72, 86, 87).

## Management of ICIs-Related Thyroid Dysfunction

In recent studies (17, 88–90), higher OS and PFS are observed among patients with thyroid dysfunction and positive auto-antibodies when they are treated with ICIs. Nevertheless, with the increasing use of immunosuppressive therapy, clinicians should identify and regulate thyroid IRAEs to prevent further rising incidence. At present, there is no prospective trial to determine the best treatment for thyroid IRAEs and the currently recommended treatment is based on the consensus of endocrinologists and guidelines (**Figure 4**) (18, 42, 91, 92).

### Surveillance

**Figure 3:**
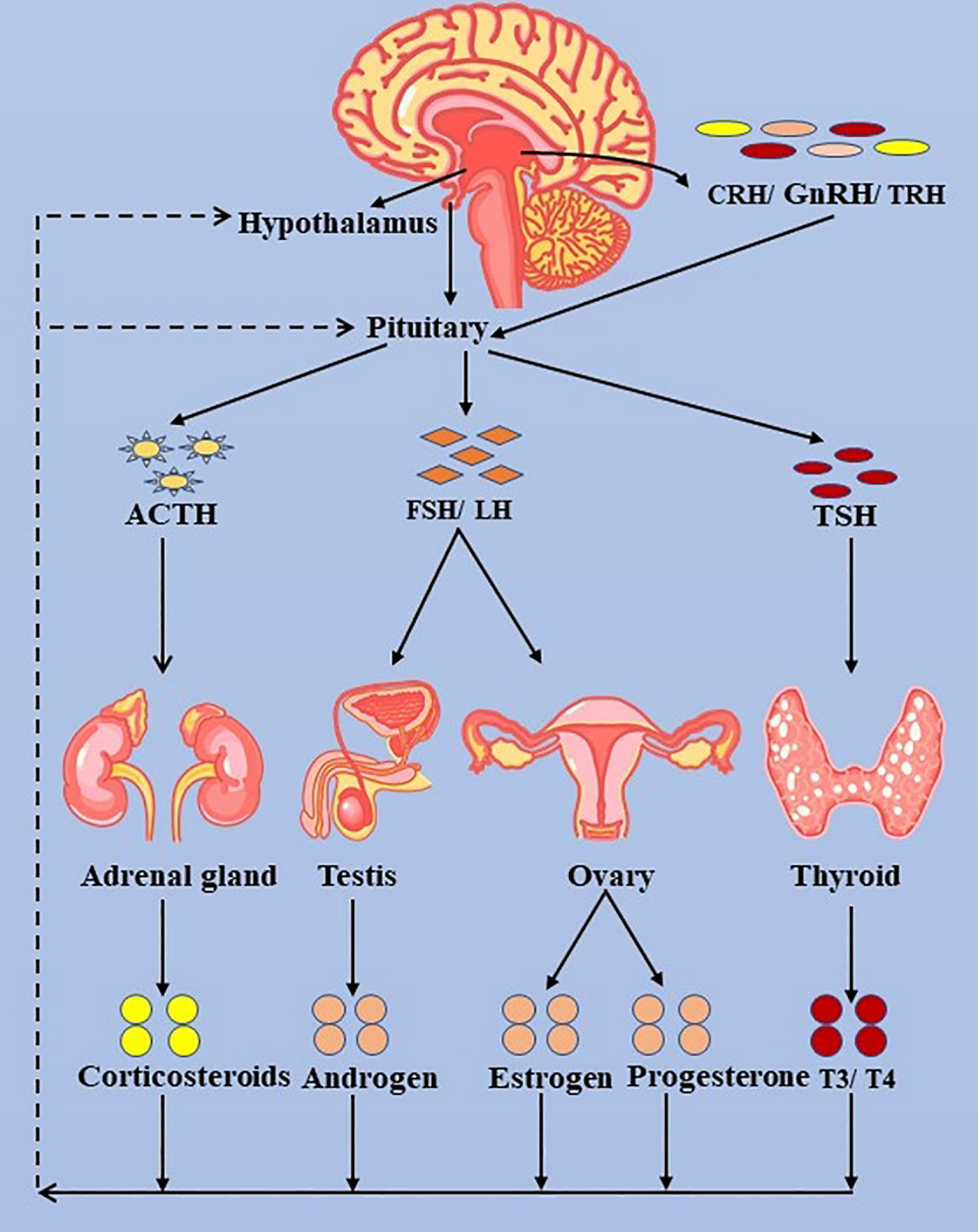
The hypothalamus-pituitary-thyroid/adrenal gland/ovary/testis axis. ACTH, adrenocorticotropic hormone; LH, luteinizing hormone; FSH, follicle-stimulating hormone; FT3, free triiodothyronine; FT4, free thyroxine; TSH, thyroid-stimulating hormone; TRH, thyrotropin-releasing hormone.

**Figure 4:**
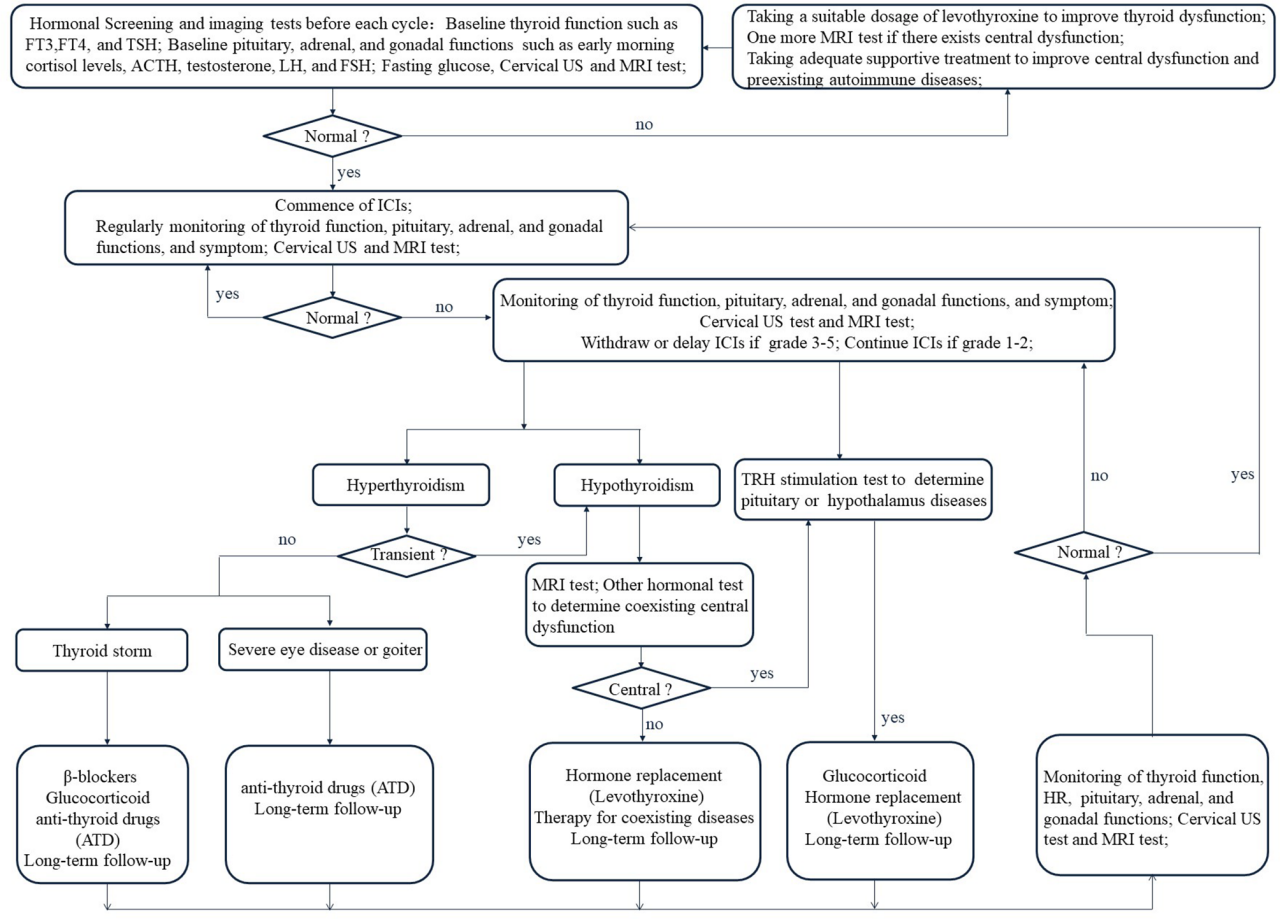
Management flow chart for Immune checkpoint inhibitors-related thyroid dysfunction. FT3, free triiodothyronine; FT4, free thyroxine; TSH, thyroid-stimulating hormone; ACTH, adrenocorticotropic hormone; LH, luteinizing hormone; FSH, follicle-stimulating hormone.

The majority of patients with thyroid dysfunction after immunotherapy are asymptomatic (18). Therefore, it is unnecessary to expect patients to have positive and marked clinical manifestations when observing ICIs-related side effects on the thyroid. However, we should routinely assess the patient’s symptoms and signs during the ICIs therapy. Moreover, we should focus on the thyroid biochemical indexes and imaging tests of patients to ascertain if it is hyperthyroidism, transient thyroiditis, or hypothyroidism, such as FT3, FT4, TSH, and cervical ultrasound (US) (92). The biochemical behavior of thyroid dysfunction is discrepant in different tumor types on specific ICIs and cycles (13). Consequently, there is no consistent conclusion on monitoring time (92, 93). Routine biochemical function tests and screening can detect endocrinopathies before symptoms appear, promoting earlier treatment and a lower incidence rate. A comprehensively initial test should include a thyroid function test (FT3, FT4, TSH), fasting glucose, pituitary functions (early morning cortisol levels), adrenal function (plasma adrenocorticotropic hormone (ACTH)), and gonadal functions (testosterone, luteinizing hormone (LH), and follicle-stimulating hormone (FSH)) (42). In addition, the possibility of central hypothyroidism should be investigated by MRI test in the case of low FT4 with low TSH, including ICIs-induced hypophysitis, and then a systematic assessment of pituitary, adrenal, and gonadal functions is required (**Figures 1**, **3**, **4**) (18, 56). It is important to check coexisting hormonal disorders and thyroid function in case of hormonal replacement therapy. Besides, long-term follow-up for thyroid IRAEs is recommended (92).

In recent years, numerous scholars are trying to explore markers related to thyroid IRAEs (71, 94). There exists controversy about the exclusion of patients with autoimmune diseases from ICI therapy. There is increasing evidence that ICIs may be safe and effective in cancer patients with preexisting autoimmune diseases (95–97). Prospective studies to testify such novel strategies among patients with autoimmune diseases are needed. Nevertheless, the guidelines suggest that thyroid disease-related symptoms and signs, thyroid function test, other hormonal function tests and imaging tests should be detected before the beginning of immunotherapy and each treatment cycle (18). It is uncertain whether baseline assessment of thyroid antibodies will help identify the risk of thyroiditis because patients with a history of autoimmune diseases are mostly excluded from clinical ICIs trials (77). Currently, although there is insufficient data to recommend routine measurement of thyroid antibodies as a baseline standard, this may be a useful follow-up test to determine those who are more likely to have a persistent disease rather than transient drug-induced thyroiditis (17, 92). Most scholars suggested that close follow-up should be performed in patients who have high TPO-Ab at baseline or a history of hypothyroidism because they believe that it indicates an increased risk of hypothyroidism deterioration after the employment of ICIs (9, 71, 75, 76). Test for TG-Ab and TSH receptor antibodies (TR-Ab) is necessary if there are clinical features and suspicions of GD (**Figure 1**) (92).

### Treatment

As aforementioned, thyroid IRAEs has a relatively consistent pattern from the initial stage of transient hyperthyroidism to hypothyroidism or direct hypothyroidism (9, 13, 98). The presence of symptoms and the biochemical confirmation of evident subclinical hypothyroidism or hypothyroidism are the indications to start continuous thyroxine treatment (49). Taking a suitable dosage of levothyroxine in solid form on an empty stomach is the main choice (42, 92). However, elderly patients and patients with heart disease should receive low-dose levothyroxine (56, 91). Transient hyperthyroidism should not be treated because it usually subsides naturally and often transforms into hypothyroidism. However, when faced with serious thyrotoxicoses, such as thyroid storm (16), severe eye disease, or goiter (99), doctors should respond swiftly to ensure that patients get the best results. Supportive therapy of β-blockers, glucocorticoid, and anti-thyroid drugs (ATD) is helpful to relieve the symptoms of serious thyrotoxicosis (92, 100). Clinicians should decide whether ICIs should be stopped or delayed after the occurrence of thyroid dysfunction based on the grade in the CTCAE Version 5.0. (**Table 2**) (58). If there are coexisting hormonal disorders with thyroid function during hormonal replacement therapy, adequate supportive treatment should be considered in clinical practice (18, 42, 91, 92). Meanwhile, the employment of ICIs should be guided based on IRAEs grade (92). ICIs should be withdrawn or delayed if thyroid or other organ IRAEs are graded from level 3 to 5 but ICIs could be continued if thyroid or other organ IRAEs are graded from level 1 to 2 (18, 42, 91, 92). Of course, in case of IRAEs with adequate supportive treatment, ICIs can be continued or restarted (18, 42, 91, 92, 101, 102).

## Conclusion

Thyroid dysfunction is the most common IRAEs, which warrants close attention from endocrinologists and oncologists. Thyroid IRAEs may involve T and B-lymphocytes, multiply cytokines, and diverse factors. With a limited understanding of the pathogenesis, it is not clear whether the immune cells responsible for IRAEs are the same as those involved in enhancing the anti-tumor immune response and HT. It is also controversial whether malignant tumor coexisting autoimmune diseases should be excluded from ICIs. We do not always exclude using ICIs for cancer patients with a preexisting autoimmune disease from the current understanding and consensus. Further clinical and laboratory researches should be conducted to improve the understanding of ICIs-related thyroid dysfunction. Additionally, the identification and management of thyroid IRAEs should be enhanced to avoid life-threatening complications and increasing mobility. Besides, the long-term effects of ICIs on thyroid function should be evaluated in future studies to better understand thyroid IRAEs and AITDs.

## Data Availability Statement

The original contributions presented in the study are included in the article/supplementary material. Further inquiries can be directed to the corresponding authors.

## Author Contributions

The manuscript was jointly written by LZ and H-FF. LZ, H-FF, X-LY, and S-RS contributed to the conception and design, the acquisition of data, the figure, and the drafting of the manuscript. H-QL, L-TG, CC, and X-LY collected and assembled the quantitative data. All authors contributed to the design and interpretation of the review, and reviewed and wrote the final paper.

## Funding

This work was supported by the National Natural Science Foundation of China (Grant number: 81302314), Hubei Provincial Natural Science Foundation (Grant number: 2019CFB303) and Hubei Provincial Key Laboratory of Occupational Hazard Identification and Control, Wuhan University of Science and Technology (Grant number: OHIC2020Z06).

## Conflict of Interest

The authors declare that the research was conducted in the absence of any commercial or financial relationships that could be construed as a potential conflict of interest.

## Abbreviations

ICIs, immune checkpoint inhibitors; IRAEs, immune-related adverse events PD-1, programmed cell death gene-1; PD-L1, programmed cell death gene-1 ligand; CTLA-4, cytotoxic T-lymphocyte-associated antigen-4; APCs, antigen-presenting cells; DCs, dendritic cells; RCT, randomized controlled trial; LUAD, lung adenocarcinoma; SCLC, small cell lung cancer; NSCLC, non-small cell lung cancer; SCCA, squamous cell carcinoma of the anal canal; cSCC, cutaneous squamous cell carcinoma; TNBC, triple-negative breast cancer; UTUC, urothelial carcinoma; Th cells, helper T cells; CTL, cytotoxic T lymphocyte cell; Treg, T-regulatory cells; AITDs, autoimmune thyroid diseases; HT, Hashimoto’s thyroiditis; GD, Graves’ disease; FT3, free triiodothyronine; FT4, free thyroxine; TSH, thyroid-stimulating hormone; TPO-Ab, thyroperoxidase antibodies; TG-Ab, thyroglobulin antibody; Tg, thyroglobulin; CTCAE, the Common Terminology Criteria for Adverse Events; HLA, human leukocyte antigen; MHC, major histocompatibility complex; ACTH, Adrenocorticotropic hormone; LH, Luteinizing hormone; FSH, Follicle-stimulating hormone; TRH, Thyrotropin-releasing hormone; TRAb, TSH receptor antibodies.
